# Acute acrylamide poisoning with severe symptoms in a short time: a case report

**DOI:** 10.1186/s12245-023-00514-z

**Published:** 2023-06-29

**Authors:** Rie Yamamoto, Takayuki Yasuoka, Junya Matsushima, Youhei Tsubouchi, Hideaki Kanazashi, Keiji Sakurai, Tomoki Hanazawa, Yoshito Kamijo, Kazuki Akieda

**Affiliations:** 1 Department of Emergency and Critical Medicine, Tokai University Hospital, 143 Shimokasuya, Isehara-shi, Kanagawa 259-1193 Japan; 2 Emergency Department, SUBARU Health Insurance Society, Ota Memorial Hospital, Oshimacho, Ota, Gunma Japan; 3 Clinical Toxicology Center, Saitama Medical University Hospital, 38 Morohongo, Moroyama-machi, Iruma-Gun, Saitama Japan

**Keywords:** Acute acrylamide poisoning, Dose, Dose rate, Severity

## Abstract

**Background:**

Acrylamide poisoning is often reported as chronic poisoning presenting with peripheral neuropathy or carcinogenic action due to long-term exposure to low concentrations. However, there have been few reports of acute poisoning due to oral ingestion of acrylamide, where the symptoms appear a few hours after ingestion. Here, we report a case of acute acrylamide poisoning where a high concentration was ingested in a short time, resulting in a fatal outcome due to the rapid course of events.

**Case presentation:**

The patient was an adolescent female who ingested 150 ml (148 g) of acrylamide with suicidal intent. A disorder of consciousness was observed when the emergency medical team arrived 36 min later. An hour later, tracheal intubation and intravenous access were performed at a hospital, and 2 h after that, she was transported to our hospital. After she arrived at the hospital, circulatory dynamics could not be maintained despite vasopressor and colloid osmotic infusion, and hemodialysis could not be introduced. Subsequently, cardiopulmonary arrest occurred, and the patient passed away 7 h after ingestion.

In the present case, severe symptoms appeared shortly after acrylamide ingestion, unlike other reported cases. In previous report summarizing animal studies, there was a relationship among the symptoms of acute poisoning, the dose, and onset time. The data from this case were compared to those from previous reports, and we were able to predict the early appearance of severe symptoms based on this comparison.

**Conclusion:**

The severity of acute acrylamide poisoning by oral ingestion was primarily dependent on the amount and rate of ingestion.

## Background

Acrylamide (CH2 = CHCONH2) is an odorless white crystalline-powdered vinyl monomer found in paper, waterproofing agents, electrophoresis gel, paint, heat processed foods, etc. [1, 2]. Acrylamide is absorbed into the body via all the oral, transdermal, and airway routes and has neurotoxic, hepatotoxic, and carcinogenic effects. In Japan, acrylamide has been designated as a deleterious substance according to the “Poisonous and Deleterious Substances Designation Order” [3].

Acrylamide is easily and rapidly absorbed from the skin, respiratory system, and gastrointestinal tract and is distributed widely in vivo [4]. It has been reported to possess genotoxic, germ cell toxicity, and carcinogenic potential. Furthermore, acrylamide affects nerve endings, thereby causing central nervous system (CNS) symptoms, including hallucinations and convulsions, and peripheral nervous system symptoms, including tremors, weakness, ataxia, and sensory abnormalities [2, 5, 6]. Almost cases of acrylamide poisoning reported to date have been of peripheral neuropathy and carcinogenic effects via ingestion of drinking water, heat-processed food, or occupational exposure. Its severity depends on the dosage, rate of exposure, and duration of exposure, with few reports on acute poisoning [7]. Futhermore, there have been only a few reports of acute poisoning due to the oral intake of acrylamide with suicidal intent were noted. Particularly, acute poisoning with suicidal intent is rarely reported with dosage. In a review of literature, there were reports of a dosage of 13 g by Joo et al. [8] in 2012, a dosage of 30 g by Mohammadi et al. [9] in 2015, a dosage of 60 g by Okuda [10] in 2019, and a dosage of 18 g by Donovan in an abstract in 1987 [11]. Although detailed clinical manifestations of acute intoxication are occasionally unclear, in these studies, gastrointestinal symptoms, including nausea and vomiting, appear relatively early after ingestion and CNS symptoms, including hallucinations, restlessness, and convulsions, appear several hours later. Moreover, only animal studies on the severity of acute acrylamide poisoning have been published.

In contrast to previous reports, we encountered a fatal case of acute acrylamide poisoning where the patient could not be saved due to the rapid progression of symptoms. Here, we report a case in which a high-concentration acrylamide solution was ingested in a short time and investigate the factors that played the primary role in the fatal outcome.

### Case presentation

The patient was an adolescent female with no past medical history who ingested 150 ml (148 g) of 99% acrylamide solution in her room with suicidal intent. She was discovered by her mother 30 min after ingestion and had collapsed and was vomiting in the corridor, and an ambulance was called. At 36 min after ingestion, when the emergency medical team arrived, the Glasgow Coma Scale (GCS) score was eye opening (E); 3, best verbal response (V); 4, best motor response (M); 5, respiratory rate was 35/min, pulse was 115/min, blood pressure was 64/23 mmHg, body temperature was 37.1℃, SpO_2_ was 75% (room air), and the pupils were at 4.0/4.0 mm. At 52 min after ingestion, she suffered a generalized convulsion in the ambulance, and her vitals subsequently deteriorated to a GCS of E1V1M4, respiratory rate of 20/min, pulse of 124/min with non-palpable blood pressure, and SpO_2_ of 96% (10 L reservoir mask [RM]).

One hour after ingestion, the patient arrived at a hospital, and the doctor started tracheal intubation, two intravenous accesses, and gastric tube insertion. Blood tests revealed elevated WBC (14,500/µL), mild renal dysfunction (Cr. 1.09 mg/dL), and metabolic acidosis (pH 7.047, PaCO_2_ 40.9 mmHg, and lactate 14.24 mmol/L). Sodium bicarbonate was administered, and pH was restored to 7.423 in approximately 15 min.

When transferred to our hospital for intensive care (2 h after ingestion), GCS was E1VTM1, respiratory rate was 17/min (assisted ventilation), pulse was 131/min, blood pressure was 68/42 mmHg, body temperature was 37.1℃, SpO_2_ was 99% (100%), pupils were 4.0/4.0 mm, and the pupillary light reflex had disappeared. Physical examination showed no cyanosis, but cold sweat was noted all over the body with vomiting marks around the mouth. Breathing sound was heard on both sides with no noise, the abdomen was soft, and there was no general edema. Blood tests revealed hypercapnia and lactic acidosis (pH 7.351, PaCO_2_ 47.1 mmHg, and lactate 8.3 mmol/L), as well as high-sensitivity cardiac troponin I elevation (321.7 pg/ml). We started respiratory management with a ventilator and circulatory management with vasopressors. At 25 min after arrival, we inserted a central venous catheter and blood access catheter to introduce continuous hemodialysis, which ultimately could not be initiated due to the progression of circulatory failure. One hour after arrival, generalized convulsions appeared; therefore, we administered sedatives and muscle relaxants. Two hours after arrival, blood tests revealed prolonged blood coagulability, in addition to hypoalbuminemia (Alb 1.3 g/dL) and elevated transaminase (AST 79 U/L, ALT 37 U/L). We administered colloid osmotic infusion, but 3 h after arrival, respiratory and lactic acidosis, and increased bicarbonate ions (pH 7.340, PaCO_2_ 53.6, HCO_3_ 28.1 mmol/L, lactate 9.3 mmol/L, and anion gap 15.9 mEq/L) due to compensatory effect appeared. And the patient’s circulation then became unsustainable. At 3.5 h after arrival, the carotid artery became non-palpable; we continued cardiopulmonary resuscitation but spontaneous heartbeat did not resume, and the patient passed away 5 h after arrival (7 h after ingestion).

### Serum acrylamide concentration

We measured the acrylamide concentration from the serum using liquid chromatography-mass spectrometry. Specimens were collected at the following three time points: immediately after arrival at our hospital, 1.5 h after arrival (when a generalized convulsion appeared) and 3.5 h after arrival (at the time of cardiopulmonary arrest). Acrylamide concentration was 801.5 µg/mL at our hospital arrival, 495 µg/mL 1.5 h after arrival, and 390.2 µg/mL 3.5 h after arrival. However, by the time the patient was transported to our hospital, 2 h had passed since the acrylamide was ingested and the initial concentration could not be measured.

## Discussion and conclusions

In this case, the patient ingested a high-concentration acrylamide solution, and the symptoms of acute poisoning appeared in a short time. In previously reported cases of acute acrylamide poisoning, central nervous symptoms appeared several hours after ingestion, in contrast to the present case, where severe symptoms appeared shortly after ingestion and the patient died. Herein, we determined that the cause of death was acute circulatory failure due to acrylamide poisoning.

According to previous reports of animal studies, the LD_50_ of acrylamide is said to be approximately 100–200 mg/kg based on animal studies [12]. In this case, body weight was not measured, but the patient had a standard physique; therefore, we assume that she was of the mean weight of Japanese women of the same age (50 kg), and the LD_50_ for the patient was calculated as 5–10 g. However, the patient ingested 148 g. The dosage far exceeded the LD_50_; therefore, the case was deemed to be one of severe poisoning.

Several toxic effects have been observed for acrylamide; however, remain unclear except for neurotoxicity. Recent studies on the neurotoxicity of acrylamide have shown that acetylcholinesterase activity is decreased in the muscle tissue and hypothalamus, indicating structural damage and nerve changes due to direct effects. It has been noted that early and progressive changes in nerve endings in the CNS region appear. Moreover, a dose-dependent increase in mortality, motor impairments, and oxidative stress has appeared. Another study analyzing acrylamide and key neurotransmitters reported CNS depression due to its effects on cholinergic, serotonergic, and dopaminergic activities, as well as a decrease in other neurotransmitters. In particular, dopamine and norepinephrine levels were significantly reduced [13, 14].

Herein, 148 g of acrylamide were ingested in a 150-mL solution. Generally, it is possible to consume 150 mL of beverage in a short period; therefore, we assumed that the patient also ingested 150 mL of solution in a short period. Therefore, we assumed that the rapid absorption of a lethal dose of acrylamide into the body and the rapid changes in the neuronal cell and developed neurotransmitters, and would have resulted in the appearance of severe central nervous symptoms such as a disorder of consciousness and convulsion, and hypotension appeared early time after ingestion.

The metabolic pathway of acrylamide has been shown in animal studies in mice and rats. Acrylamide absorbed in the body is detoxified by glutathione conjugation in the liver to *N*-acetyl-S-(2-carbamoyl ethyl)cysteine and excreted in the urine. In another pathway, the epoxide glycidamide is formed by cytochrome P450 phenotype 2E1. Glycidamide is excreted in the urine by glutathione conjugation to *N*-acetyl-S-(2-carbamoyl-2-hydroxyethyl)cysteine and *N*-acetyl-S-(1-carbamoyl-2-hydroxyethyl)cysteine [15, 16]. In this case, no significant hepatic dysfunction was observed; therefore, symptomatic treatment was not administered.

Acrylamide slows or inhibits glycolytic metabolism by inducing changes in the tricarboxylic acid (TCA) cycle and glycolytic proteins. Furthermore, it attenuates the activity of the mitochondrial electron transfer chain complex and increases oxidative stress [17]. In this case, hyperlactatemia was observed, and it was assumed that glycolytic system inhibition promoted anaerobic metabolism, thereby leading to progressive metabolic acidosis and the appearance of tachycardia and tachypnea. However, we also considered the possibility that convulsions in the ambulance may have affected metabolic acidosis.

In addition, in a report by Hashimoto summarizing previous reports of animal studies on the relationship between the symptoms of acute poisoning, the dose, and onset time relationship, when 65 mg/kg of acrylamide was administered to cats, central nervous symptoms appeared after several hours, and with 100 mg/kg, convulsion appeared and some died after 10 h. Furthermore, symptoms appeared and all animals died within several hours with 200 mg/kg, in 2–3 h with 500 mg/kg, and within an hour with 1000 mg/kg [12]. Assuming that the body weight of this present case was 50 kg, the dosage of 148 g is equivalent to 2960 mg/kg. Even though the subjects were different, when simply comparing values, the patient ingested a dose of about three times 1000 mg/kg, at which dose fatal symptoms appear within an hour. Based on the relationship between the symptoms of acute poisoning, the dose, and the onset time relationship, it could have been possible to predict the severe and lethal course of the case. However, there have been reports of patients ingesting more than 1000 mg/kg, but symptoms appeared after over half a day, and the patients were finally saved [10].

In general, the organs involved in drug metabolism and excretion are the gastrointestinal tracts, liver, and kidneys. Gastric pH and gastric emptying time, drug metabolizing enzymes in the gastrointestinal tract and liver, transporters in the liver, and glomerular filtration rate in the kidney are said to develop with age [18–20]. Therefore, the half-life is longer in childhood than that in adulthood [19]. The present case was in the transition period from childhood to adulthood, and it is assumed that the gastrointestinal tract, liver, and kidneys were already functioning as well as those of an adult. However, since drug absorption, metabolism, and excretion vary with age, the possibility cannot be ruled out that the age at which acrylamide is ingested may affect the severity of the disease.

This case has shown a course consistent with the report by Hashimoto, and it could be very likely that the severity of acute acrylamide poisoning was also affected by dosage and rate of administration. However, for suicide intent, acrylamide was ingested in large amount at one time; therefore, it is difficult to evaluate the association of the severity of poisoning with the duration of exposure.

Absorption processes and excretion rates are significant in pharmacokinetics and toxicokinetics [21, 22]. To consider the toxicokinetics of this study, we used serum acrylamide concentrations at the time of hospital arrival, 1.5 h later, and 3.5 h later. From these periods, we derived an elimination rate constant of 0.18 and a further half-time of 3.85 for this case. Some studies have reported a half-time of 5 h for acrylamide [10], and the present result was slightly shorter at 3.8 h. However, in the present case, concentrations immediately after administration and at 1 h were unavailable, making it challenging to accurately assess and predict peak serum concentrations and the toxicokinetics of acrylamide. Glycidamide, a metabolite of acrylamide, has a smaller molecular weight and a longer half-life than acrylamide [10]. Additionally, we considered measuring serum glycidamide levels; however, we were unable to do so in this case. To clarify the association between changes in acrylamide and glycidamide concentrations and changes in physical symptoms, further studies are needed.

Other factors affecting acrylamide severity, the metabolism of acrylamide, and treatment methods are not clear and need to be clarified through future studies. This may lead to the development of early measures for treating exacerbation and optimal treatments for poisoning based on the symptoms present. Acrylamide poisoning is rare; however, when a large amount of acrylamide is ingested, as in this one, the patient can become severely ill in a short period. Currently, symptomatic treatment is the mainstay of treatment; however, it is recommended that the patient be promptly transported to a medical institution that has a regular supply of *N*-acetylcysteine and introduce dialysis at an early time and immediately treat the patient. This will help save patients with acrylamide poisoning even after the ingestion of high-dose acrylamide.

Based on this case study, the severity of acute acrylamide poisoning by oral ingestion is primarily dependent on the amount and rate of ingestion.

## Data Availability

The datasets used and/or analyzed during the current study are available from the corresponding author on reasonable request.

## Abbreviations

GCS: Glasgow coma scale
E: Eye opening
V: Best verbal response
M: Best motor response
RM: Reservoir mask
LD_50_: 50% Lethal dose
TCA: Tricarboxylic acid
